# Impact of a publicly-funded pharmacare program policy on benzodiazepine dispensing among children and youth: a population-based natural experiment

**DOI:** 10.1186/s12887-023-04331-4

**Published:** 2023-10-19

**Authors:** Tony Antoniou, Daniel McCormack, Sophie Kitchen, Kathleen Pajer, William Gardner, Yona Lunsky, Melanie Penner, Mina Tadrous, Muhammad Mamdani, David N. Juurlink, Tara Gomes

**Affiliations:** 1 Li Ka Shing Knowledge Institute, St. Michael’s Hospital, Toronto, ON Canada; 2 ICES, Toronto, ON Canada; 3 Department of Family and Community Medicine, University of Toronto, Toronto, ON Canada; 4 Department of Family and Community Medicine, St. Michael’s Hospital, Toronto, ON Canada; 5 Children’s Hospital of Eastern Ontario Research Institute, Ottawa, ON Canada; 6 Department of Psychiatry, University of Ottawa, Ottawa, ON Canada; 7 School of Epidemiology and Public Health, University of Ottawa, Ottawa, ON Canada; 8 Azrieli Adult Neurodevelopmental Centre, Centre for Addiction and Mental Health, Toronto, Canada; 9 Department of Psychiatry, University of Toronto, Toronto, ON Canada; 10 Autism Research Centre, Bloorview Research Institute, Holland Bloorview Kids Rehabilitation Hospital, Toronto, Canada; 11 Department of Pediatrics, University of Toronto, Toronto Ontario, Canada; 12 Leslie Dan Faculty of Pharmacy, University of Toronto, Toronto, ON Canada; 13 Li Ka Shing Centre for Healthcare Analytics Research & Training, Unity Health, Toronto, ON Canada; 14 Temerty Faculty of Medicine, University of Toronto, Toronto, ON Canada; 15 Institute of Health Policy, Management, and Evaluation (Mamdani), University of Toronto, Toronto, ON Canada; 16 Department of Medicine, University of Toronto, Toronto, ON Canada

**Keywords:** Child, Adolescent, Benzodiazepines, Policy, Prescriptions / statistics & numerical data

## Abstract

**Background:**

In January 2018, the Government of Ontario, Canada, initiated a universal pharmacare program (OHIP+) for all individuals aged 24 years and younger. In April 2019, the program was amended to cover only children and youth without private insurance. Because benzodiazepines are commonly prescribed to children and youth despite their potential hazards, we examined whether changes in publicly-funded drug coverage influenced benzodiazepine dispensing trends in this demographic.

**Methods:**

We conducted a population-based natural experiment study of benzodiazepine dispensing to children and youth in Ontario between January 2013 and March 2020. We used interventional autoregressive integrated moving average models to estimate the impact of OHIP + and its subsequent modification on these trends.

**Results:**

The implementation of OHIP + was associated with an immediate increase in the monthly rate of benzodiazepine dispensing of 12.9 individuals per 100,000 population (95% confidence interval [CI]; 7.5 to 18.3 per 100,000). Benzodiazepine dispensing rates rose from 214.2 to 241.5 per 100,000 from December 2017 to March 2019, a 12.8% (95% CI 9.6–16.0%) increase. In stratified analyses, increases were most pronounced among females, children and youth living in the lowest income neighbourhoods and individuals aged 20 to 24. The April 2019 modification to OHIP + was not associated with changes in monthly benzodiazepine dispensing trends (0.39 individuals per 100,000; 95% CI -1.3 to 2.1 per 100,000). However, rates remained elevated relative to the period preceding OHIP + implementation.

**Conclusions:**

Implementation of a publicly-funded pharmacare program resulted in more children and youth being prescribed benzodiazepines.

**Supplementary Information:**

The online version contains supplementary material available at 10.1186/s12887-023-04331-4.

## Introduction

The use of psychotropic medications in children and youth has increased worldwide [1]. However, in contrast to stimulants, antipsychotics, and antidepressants, little is known about patterns of benzodiazepine use in children and youth [1]. Studies from several jurisdictions in Europe and North America with publicly funded healthcare or combined private and public healthcare systems have found that the prevalence of benzodiazepine dispensing in children and youth ranges from 0.2 to 9.8%, with use increasing over time [1–8]. In a study from Manitoba, Canada, the incidence of benzodiazepine use among children and adolescents aged 17 and under increased from 2.2 to 3.95 per 1000 population between 1996/97 and 2011/12 [7]. In addition, findings from Ontario, Canada indicate that increased use in children and youth may be associated with harm. Specifically, between 2013 and 2020, rates of benzodiazepine-related toxicity healthcare encounters increased from 11.1 to 16.0 per 100,000 and 39.9 to 66.6 per 100,000 among children and adolescents under the age of 18 and youth aged 19 to 24, respectively [9].

While increased benzodiazepine use may reflect improved recognition and diagnosis of anxiety disorders in children and youth, there is a lack of research supporting the effectiveness and long-term safety of benzodiazepines in this population [10, 11]. Consequently, benzodiazepines are not approved as anxiolytics for children in North America or Europe, with clinical practice guidelines preferentially advocating behavioural therapies over medication [12, 13]. Moreover, although these drugs are sometimes used short-term in combination with antidepressants approved for anxiety disorder, [14] this practice is not recommended in individuals below the age of 18, and benzodiazepines are not endorsed as a therapeutic option for any psychiatric disorder in this population [12, 13]. In light of the increasing off-label use of benzodiazepines in children and youth and concerns about the possibilities of dependence, diversion and nonmedical misuse, [15–19] research examining patterns of pediatric benzodiazepine use and the influence of specific interventions on the use of these drugs is needed to inform clinical practice and policy.

Although several studies have examined the impact of policies and guidance intended to curb benzodiazepine use among adults, [20–22] less is known about the influence on benzodiazepine use of interventions intended to increase access to prescription medications more generally. In January 2018, the Ontario government implemented a publicly-funded pharmacare program known as OHIP + to provide all Ontarians aged 24 and younger prescription medications listed on the Ontario Drug Benefit formulary at no cost [23]. Coverage was automatic, with no deductibles or copayments. The program was subsequently modified in March 2019 to only cover medications for children and youth without private insurance [24]. Because OHIP + eliminated out-of-pocket costs and disparities in drug insurance coverage, we postulated that dispensing of benzodiazepines to children and youth may have increased immediately following implementation. Additional changes in benzodiazepine use may have also occurred with the subsequent modification of OHIP + in April 2019, which restricted universal drug coverage to children and youth without private insurance. Therefore, the implementation and modification of OHIP + offered a natural experiment for evaluating the influence of universal pharmacare on the use of benzodiazepines in children and youth and evaluating the differential impact of these policy changes on various populations of children and youth. Accordingly, we studied the impact of OHIP + and its modification on benzodiazepine dispensing among the entire population of eligible individuals aged 24 and younger in Ontario, home to approximately 40% of Canadian children and youth [25]. We speculated that the implementation of OHIP + would be associated with an increase in benzodiazepine use and that a more gradual change would be observed following its modification to cover only those children without private insurance.

## Methods

### Setting

We conducted a population-based study of all residents in Ontario aged 0 to 24 years between January 1, 2013, and March 31, 2020.

### Data sources

We used Ontario’s administrative health databases. These datasets were linked using unique encoded identifiers and analyzed at ICES (formerly known as the Institute for Clinical Evaluative Sciences). We identified prescriptions for benzodiazepines using the Narcotics Monitoring System database, which contains comprehensive records of all prescriptions for controlled substances dispensed from community pharmacies in Ontario, regardless of payer. Because we were focusing on the use of benzodiazepines for mental health conditions, we excluded prescriptions for clobazam, which is used primarily for seizure disorders. We used the Registered Persons Database, a registry of all individuals eligible for the publicly-funded Ontario Health Insurance Plan, to ascertain demographic characteristics for all children and youth dispensed benzodiazepines over the study period. We determined the proportions of individuals diagnosed with anxiety, mood, or seizure disorders within 30 days prior to or on the prescription dispensing date using outpatient physician claims data from the Ontario Health Insurance Plan database, emergency department data from the Canadian Institute for Health Information National Ambulatory Care Reporting System, and hospitalization data from the Canadian Institute for Health Information Discharge Abstract Database and Ontario Mental Health Reporting System (see supplemental Table 1 for diagnostic codes). The OHIP database includes all physician claims (primary care and specialist) for medical services covered under the provincial health insurance plan. We obtained prescriber specialty information using the ICES Corporate Provider Database. The use of data in this project was authorized under Sect. 45 of Ontario’s Personal Health Information Protection Act, which does not require review by a Research Ethics Board, or individual patient consent. ICES approved the study and all analyses of raw data were conducted at ICES. No other administrative permissions were required.

### Study Population and Outcomes

For each month in the study period, we defined our study population as all Ontario residents aged 0 to 24 who were alive on the first day of the month. Our primary outcome was the monthly rate of benzodiazepine use per 100,000 children and youth, defined as the number of individuals dispensed a benzodiazepine in a given month divided by the population of children and youth aged 0 to 24 for that period. To determine whether characteristics of children and youth dispensed a benzodiazepine changed following the implementation and modification of OHIP+, we compared demographic characteristics and prescriber type among individuals receiving benzodiazepines during the period immediately preceding the implementation of OHIP+ (January 1, 2017, to December 31, 2017), during OHIP+ (January 2018 to March 2019) and the period in which OHIP + was available only for children and youth without private insurance (April 2019 to March 2020).

### Statistical analysis

We used interventional autoregressive integrated moving average (ARIMA) models to examine the impact of the implementation of OHIP + and its subsequent modification on benzodiazepine dispensing rates among children and youth [26, 27]. We used the Dickey-Fuller test to determine the stationarity of the time series and applied first order and seasonal differencing to arrive at a stationary series if needed [27, 28]. The Dickey-Fuller statistic tests the null hypothesis a unit root is present in a time series and that the time series is non-stationary (i.e. does not exhibit constant variance over time) against the alternative hypothesis of stationarity. A p-value of greater than 0.05 and a failure to reject the null hypothesis indicates the presence of a unit root and that differencing is required to render the time series stationary. We used the autocorrelation function and partial autocorrelation function to identify autoregressive and/or moving average components in each time series and correct for any autocorrelation remaining after differencing, and selected the best models using goodness of fit tests [26, 27]. We used residual plots and the Portmanteau statistic to confirm that residuals from specified ARIMA models were a white noise process [29]. The null hypothesis of the Portmanteau test is that there is no residual autocorrelation and that the residuals are a white noise process. A p-value of greater than 0.05 is therefore required to infer the lack of residual autocorrelation before the ARIMA model can be used for inference and forecasting. Finally, once the ARIMA models were specified, we used a step intervention function to test for a change in the rate of benzodiazepine dispensing during the period in which OHIP + provided universal coverage of prescription medication to Ontario children and youth (January 1, 2018 to March 31, 2019), and compared predicted values of dispensing during this period with the observed rates [26, 27]. The ARIMA model also included a ramp intervention function to determine if benzodiazepine dispensing rates changed following modification to the program in April 2019, providing coverage only for children and youth with no private insurance. To explore heterogeneity in the impact of the OHIP + program, we stratified our analyses by sex, age category (0 to 9 years, 10 to 14 years, 15 to 19 years, 20 to 24 years), neighbourhood income quintile and urban versus rural residence, defined on the first day of the month of interest. We used standardized differences to compare demographic characteristics between individuals receiving a benzodiazepine during the pre-OHIP+, OHIP + and modified OHIP + periods, with differences greater than 0.1 representing an imbalance between the two groups [30]. The analyses used SAS Enterprise Guide, version 7.1 (SAS Institute Inc., Cary, NC, USA).

## Results

There were 218,299 children and youth aged 0 to 24 that received a benzodiazepine prescription between January 1, 2013 and March 31, 2020. The median age of benzodiazepine-treated children and youth was 20 years (interquartile range 17 to 22 years), and the majority (n = 136,091; 62.3%) were female (Table 1). General practitioners accounted for most benzodiazepine prescribing (n = 135,218; 61.9%), with fewer children and youth receiving these drugs from pediatricians (n = 10,470; 4.8%) and psychiatrists (n = 31,863; 14.6%) (Table 1). The majority (n = 148,892; 68.3%) of individuals were dispensed a short-term (i.e. 14 days or less) supply of benzodiazepines, with nearly 1 in 5 receiving a supply exceeding 30 days in duration. The number of children with a diagnosis of anxiety, mood and seizure disorder in the 30 days prior to receiving a benzodiazepine prescription was 102,397 (46.9%), 26,599 (12.2%) and 8,817 (4.0%), respectively. Relative to individuals dispensed less than a 30 days’ supply, those dispensed more than a 30 days’ supply of were more likely to be between the ages of 20 and 24 (55.9% vs. 51.1%; standardized difference [SD] = 0.10), receive their prescription from a psychiatrist (30.9% vs. 11.4%; SD = 0.49) and have a mood disorder in the 30 days prior to receiving a benzodiazepine (18.1% vs. 11.0%; SD = 0.34) (supplemental Table 2). Overall, the demographic characteristics of children and youth receiving a benzodiazepine did not change appreciably between the pre-OHIP+, OHIP + and modified-OHIP + periods (Table 1).

**Table 1:** Demographic and clinical characteristics of individuals aged 0 to 24 dispensed a benzodiazepine, January 2013 to March 2020*

Variable^a^	Entire Study Period (January 1, 2013 to March 31, 2020)	Pre-OHIP+ (January 1, 2017 to December 31, 2017)	During OHIP+ (January 1, 2018 to March 30, 2019)	Post-OHIP+ (April 1, 2019 to March 31, 2020)
Number of individuals	218,299	49,873	62,036	49,649
Age (median, IQR)	20 (17-22)	20 (17-22)	20 (17-22)	20 (17-22)
0-4	4,279 (2.0%)	816 (1.6%)	1,128 (1.8%)	1,005 (2.0%)
5-9	6,643 (3.0%)	1,505 (3.0%)	1,871 (3.0%)	1,619 (3.3%)
10-14	17,134 (7.8%)	3,514 (7.0%)	4,419 (7.1%)	3,679 (7.4%)
15-19	76,981 (35.3%)	15,609 (31.3%)	19,344 (31.2%)	15,270 (30.8%)
20-24	113,262 (51.9%)	28,429 (57.0%)	35,274 (56.9%)	28,076 (56.5%)
Female, No. (%)	136,091 (62.3%)	31,522 (63.2%)	39,383 (63.5%)	31,441 (63.3%)
Income quintile				
1 (lowest)	42,314 (19.4%)	9,863 (19.8%)	12,108 (19.5%)	9,693 (19.5%)
2	40,657 (18.6%)	9,324 (18.7%)	11,561 (18.6%)	9,135 (18.4%)
3	41,675 (19.1%)	9,475 (19.0%)	11,878 (19.1%)	9,499 (19.1%)
4	44,379 (20.3%)	9,813 (19.7%)	12,573 (20.3%)	10,083 (20.3%)
5	49,274 (22.6%)	11,398 (22.9%)	13,916 (22.4%)	11,239 (22.6%)
Residence				
Urban	196,653 (90.1%)	45,153 (90.5%)	55,974 (90.2%)	44,750 (90.1%)
Rural	21,646 (9.9%)	4,720 (9.5%)	6,062 (9.8%)	4,899 (9.9%)
Prescriber Type				
General Practitioner	135,218 (61.9%)	30,417 (61.0%)	37,380 (60.3%)	29,288 (59.0%)
Pediatrician	10,470 (4.8%)	2,606 (5.2%)	3,196 (5.2%)	2,689 (5.4%)
Psychiatrist	31,863 (14.6%)	9,139 (18.3%)	10,953 (17.7%)	8,974 (18.1%)
Other	40,748 (18.7%)	7,711 (15.5%)	10,507 (16.9%)	8,698 (17.5%)
Average days’ supply of prescription (mean, SD)	12.30 ± 11.91	14.62 ± 14.09	13.45 ± 12.82	13.79 ± 13.71
Days’ supply category				
1 to 7	99,221 (45.5%)	19,774 (39.6%)	26,492 (42.7%)	21,156 (42.6%)
8 to 14	49,671 (22.8%)	10,956 (22.0%)	13,798 (22.2%)	10,959 (22.1%)
15 to 29	33,985 (15.6%)	8,343 (16.7%)	9,770 (15.7%)	7,844 (15.8%)
> 30	35,411 (16.2%)	10,800 (21.7%)	11,976 (19.3%)	9,675 (19.5%)
Diagnosis in 30 days preceding dispensing date				
Anxiety disorder	102,397 (46.9%)	21,748 (43.6%)	27,374 (44.1%)	21,049 (42.4%)
Mood disorder	26,599 (12.2%)	6,219 (12.5%)	7,826 (12.6%)	6,023 (12.1%)
Seizure disorder	8,817 (4.0%)	2,058 (4.1%)	2,773 (4.5%)	2,572 (5.2%)

* Based on a first prescription claim in each period, such that individuals are counted only once in the overall column but up to 3 times total in the sub-periods

^a^ Standardized differences for all variables between all treatment periods were less 0.1

The most frequently prescribed benzodiazepines during the study period were clonazepam and lorazepam, with variation observed according to age and neighbourhood income quintile. Specifically, lorazepam and clonazepam represented 56.5% and 28.0% of prescriptions dispensed to children under the age of 12, while respective estimates for those 12 years and older were 42.7% and 42.5% (supplemental Table 3). This pattern was consistent across the pre-OHIP+, OHIP + and modified OHIP + periods (supplemental Table 3). In terms of socioeconomic status, clonazepam was the most frequently dispensed benzodiazpine to children and youth in the lowest income neighbourhoods, representing 45.9% of benzodiazepine prescriptions during the study period (supplemental table 4a). The proportion of prescriptions that were clonazepam declined as neighbourhood income quintile increased, representing 38.9% of benzodiazepine prescriptions in the highest income neighbourhoods. The opposite pattern was observed for lorazepam, which accounted for 47.5% and 38.0% of benzodiazepine prescriptions in the highest and lowest income neighbourhoods, respectively (supplemental table 4a). Other benzodiazepines for which income gradients were observed included alprazolam, which was more common in the highest- relative to the lowest-income neighbourhoods (4.3% versus 3.2%), and diazepam, which was more frequently dispensed among individuals in the lowest relative to the highest income neighbourhoods (7.3% versus 4.7%) (supplemental table 4a). These patterns were consistent across the three study periods (supplemental tables 4b, 4c and 4d).

### Change in benzodiazepine dispensing rates following implementation of OHIP+

The average monthly percent change in benzodiazepine dispensing in the period preceding OHIP + was 0.3% (0.3–0.4%). We observed a modest relative percent increase in benzodiazepine dispensing to children and youth following the implementation of OHIP+, with rates increasing 12.8% (95% confidence interval [CI] 9.6–16.0%) between December 2017 and March 2019 (214.2 vs. 241.5 per 100,000 population, respectively) (Table 2). In stratified analyses, the increase was more pronounced in females (255.5 vs. 295.5 individuals per 100,000 population) than males (174.9 vs. 190.3 individuals per 100,000 population), with relative percent increases of 15.7% (95% CI 11.4–20.0%) and 8.8% (95% CI 4.0–13.7%), respectively, between December 2017 and March 2019. In addition, individuals in the lowest income neighbourhoods had the greatest relative percent increase in benzodiazepine dispensing, with rates increasing 15.5% (95% CI 8.4–22.9%) (223.0 vs. 257.6 individuals per 100,000 population) and 19.6% (95% CI 11.9–27.6%) (215.8 vs. 258.2 individuals per 100,000 population) in the lowest and second-lowest income quintile areas, respectively. Conversely, rates increased only 5.2% (95% CI -1.3–11.8%) in the highest income neighbourhoods (224.8 vs. 236.4 individuals per 100,000 population). Furthermore, individuals aged 20 to 24 (581.6 vs. 675.9 individuals per 100,000 population) and 15 to 19 (303.1 vs. 326.2 individuals per 100,000 population) had the greatest increase in benzodiazepine dispensing following the implementation of OHIP+, corresponding to relative percent increases of 16.2% (95% CI 12.0–20.5%) and 7.6% (95% CI 1.9–13.9%), respectively, between December 2017 and March 2019.

**Table 2:** Changes in benzodiazepine dispensing to children and youth following the introduction of the OHIP+ pharmacare program in January 2018

Stratification	Rate of benzodiazepine dispensing (individuals per 100,000) December 2017	Rate of benzodiazepine dispensing (individuals per 100,000 March 2019	Relative percent change, December 2017 to March 2019 (95% confidence interval	ARIMA Model	January 2018 Step Intervention Estimate (95% confidence interval)
**Overall**	214.2	241.5	12.8% (9.6 to 16.0%)	(2,1 12,0) no intercept	12.9 (7.5 to 18.3)
**Sex**					
Female	255.5	295.5	15.7% (11.4 to 20.0%)	(2,1 12,0) no intercept	16.5 (8.3 to 24.7
Male	174.9	190.3	8.8% (4.0 to 13.7%)	(3,1 12,0) no intercept	9.3 (5.2 to 13.4)
**Age**					
0 to 9	21.7	24.8	14.7% (-1.6 to 32.3%)	(3,1 12,0) no intercept	1.1 (-0.3 to 2.4)
10 to 14	62.4	67.2	7.6% (-4.9 to 21.0%)	(2,1 12,0) no intercept	-0.56 (-4.8 to 3.6)
15 to 19	303.1	326.2	7.6% (1.9 to 13.9%)	(2,1 12,0) no intercept	16.5 (7.0 to 26.0)
20 to 24	581.6	675.9	16.2% (12.0 to 20.5%)	(2,1 12,0) no intercept	41.1 (26.0 to 56.1)
**Income quintile**					
Quintile 1 (lowest)	223.0	257.6	15.5% (8.4 to 22.9%)	(0,1 12,1) no intercept	14.7 (9.0 to 20.5)
Quintile 2	215.8	258.2	19.6% (11.9 to 27.6%)	(2,1 12,0) no intercept	15.9 (8.4 to 23.4)
Quintile 3	208.8	231.6	10.9% (3.8 to 18.3%)	(3, 1 12,0) no intercept	7.1 (1.7 to 12.5)
Quintile 4	201.1	229.1	13.9% (6.6 to 21.5%)	(2,1 12,0) no intercept	16.5 (7.0 to 26.0)
Quintile 5 (highest)	224.8	236.4	5.2% (-1.3 to 11.8%)	(2,1 12,0) no intercept	14.1 (5.3 to 22.9)
**Rural versus urban residence**					
Rural	212.2	229.6	8.2% (-1.7 to 18.6%)	(0,1 12,3) no intercept	14.6 (4.6 to 24.6)
Urban	215.0	243.4	13.2% (9.9 to 16.7%)	(2,1 12,0) no intercept	12.4 (7.0 to 17.8)

**Fig. 1:**
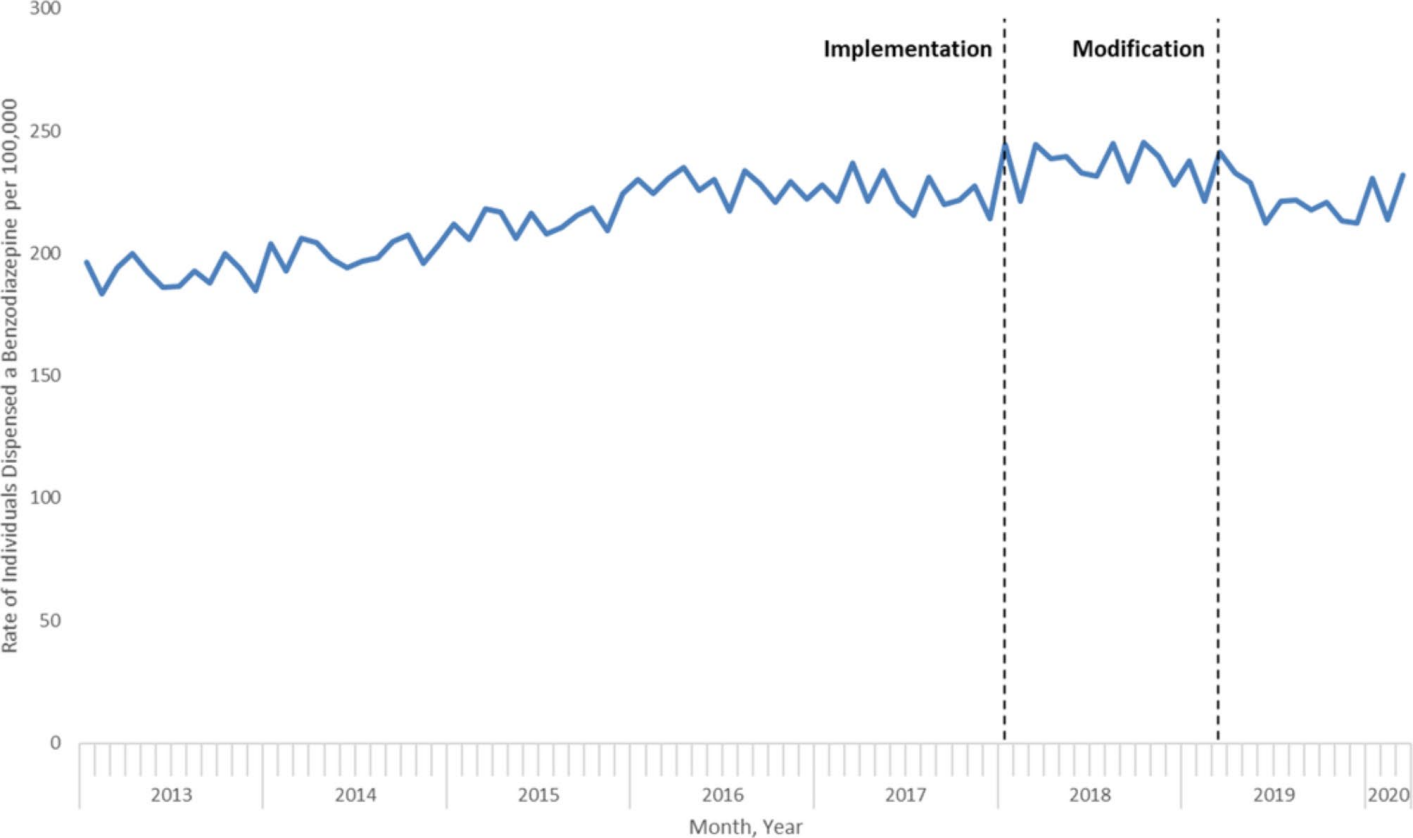
Impact of OHIP + implementation (January 2018) and modification (April 2019) on monthly rates of benzodiazepine dispensing among Ontario residents between the ages of 0 and 24, January 2013 to March 2020

Following ARIMA modelling, implementation of OHIP + was associated with a significant immediate increase in the monthly rate of benzodiazepine dispensing of 12.9 per 100,000 population (95% CI; 7.5 to 18.3 per 100,000). (Table 2; Fig. 1). The monthly rate of increase in benzodiazepine dispensing prior to OHIP + was 0.77 per 100,000 population (95% CI; 0.59 to 0.94). We observed similar results in stratified analyses (supplemental Fig. 1 to 4), with the largest immediate increases among females (16.5 per 100,000 population; 95% CI 8.3 to 24.7), individuals aged 15 to 19 (16.5 per 100,000 population; 95% CI 7.0 to 26.0) and 20 to 24 (41.1 per 100,000 population; 95% CI 26.0 to 56.1), and children and youth living in rural areas (14.6 per 100,000 population; 95% CI 4.6 to 24.6) (Table 2).

**Fig. 2:**
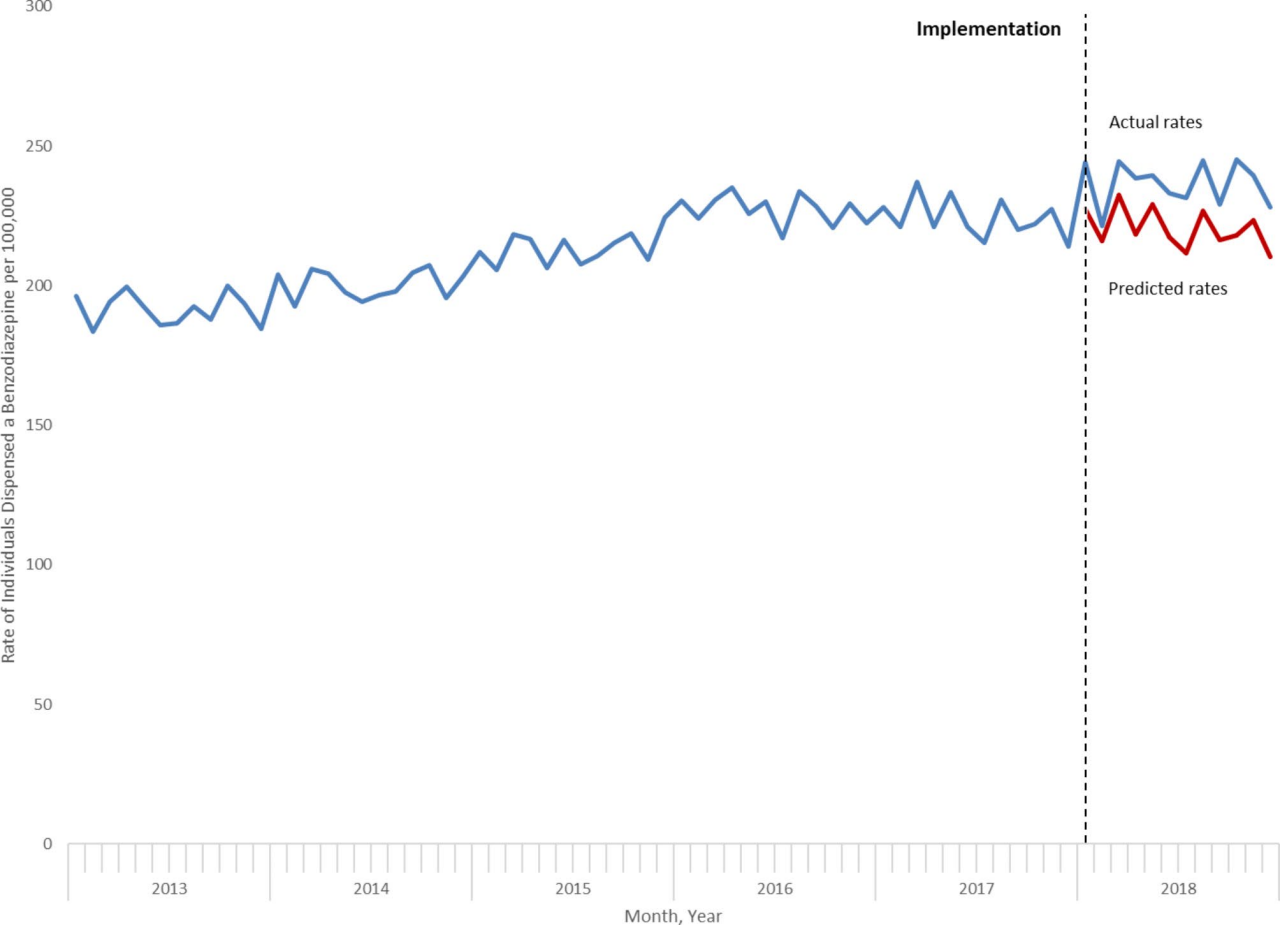
Actual versus predicted rates of benzodiazepine dispensing during OHIP+ (January 2018 to December 2018)

Observed benzodiazepine dispensing rates in the first 12 months of OHIP + were higher than those predicted by the ARIMA model in the absence of this program (Table 3; Fig. 2). The largest difference was observed in October 2018, corresponding to an additional 1,119 children and youth dispensed a benzodiazepine (Table 3).

**Table 3:** Projected and Actual Benzodiazepine Dispensing in Children and Youth, January 2018 to December 2018

Month	Projected rate (individuals per 100,000) of benzodiazepine dispensing in absence of OHIP+ (95% confidence interval)	Actual rate (individuals per 100,000) of benzodiazepine dispensing following OHIP+	Estimated absolute increase in number of children and youth dispensed benzodiazepines
January 2018	227.3 (215.1 to 239.5)	244.3	697
February 2018	216.2 (204.0 to 228.4)	221.4	212
March 2018	232.5 (219.0 to 246.1)	244.5	490
April 2018	218.3 (203.3 to 233.3)	238.6	832
May 2018	229.0 (213.7 to 244.3)	239.7	438
June 2018	217.4 (200.9 to 233.9)	233.0	639
July 2018	211.8 (194.6 to 229.0)	231.4	806
August 2018	226.8 (209.0 to 244.5)	244.7	737
September 2018	216.3 (197.8 to 234.9)	229.3	532
October 2018	218.0 (198.8 to 237.2)	245.1	1,119
November 2018	223.4 (203.5 to 243.2)	239.5	664
December 2018	210.3 (189.9 to 230.8)	228.1	733

### Change in benzodiazepine dispensing rates following modification of OHIP+

Following modification of the OHIP + program, there was a small relative percent decrease in benzodiazepine dispensing, with rates decreasing 4.0% (95% CI -6.7% to -1.3%) between March 2019 and March 2020 (241.5 vs. 231.9 individuals per 100,000 population) (Table 4). However, rates remained elevated relative to the period preceding OHIP + implementation. Following ARIMA modelling, there was no significant change in monthly benzodiazepine dispensing trends following the April 2019 modification to OHIP+ (0.39 per 100,000; 95% CI -1.3 to 2.1 per 100,000) (Table 3; Fig. 1), with similar results obtained in stratified analyses (Table 4, supplemental Fig. 1 to 4).

**Table 4:** Changes in benzodiazepine dispensing to children and youth following the April 2019 modification of the OHIP+ pharmacare program restricting coverage only to individuals without private insurance

Stratification	Rate of benzodiazepine dispensing (individuals per 100,000) March 2019	Rate of benzodiazepine dispensing (individuals per 100,000 March 2020	Relative percent change, March 2019 to March 2020 (95% confidence interval	ARIMA Model	April 2019 Ramp Intervention Estimate (95% confidence interval)
**Overall**	241.5	231.9	-4.0% (-6.7% to -1.3%)	(2,1 12,0) no intercept	0.39 (-1.3 to 2.1)
**Sex**					
Female	295.5	286.1	-3.2% (-6.7% to 0.4%)	(2,1 12,0) no intercept	0.47 (-2.2 to 3.1)
Male	190.3	180.4	-5.2% (-9.4% to -1.0%)	(3,1 12,0) no intercept	0.29 (-1.0 to 1.5)
**Age**					
0 to 9	24.8	25.6	3.0% (-11.2 to 18.2%)	(3,1 12,0) no intercept	0.18 (-0.20 to 0.60)
10 to 14	67.2	65.1	-3.1% (-14.3% to 8.7%)	(2,1 12,0) no intercept	-0.21 (-1.6 to 1.2)
15 to 19	326.2	299.9	-8.0% (-13.0% to -3.0%)	(2,1 12,0) no intercept	0.61 (-2.5 to 3.7)
20 to 24	675.9	655.8	-3.0% (-6.4% to 0.5%)	(2,1 12,0) no intercept	0.98 (-3.9 to 5.9)
**Income quintile**					
Quintile 1 (lowest)	257.6	246.2	-4.4% (-10.2% to 1.6%)	(0,1 12,1) no intercept	0.31 (-1.2 to 1.8)
Quintile 2	258.2	241.5	-6.4% (-12.3% to -0.4%)	(2,1 12,0) no intercept	-0.02 (-2.5 to 2.5)
Quintile 3	231.6	220.0	-5.0% (-11.0% to 1.2%)	(3, 1 12,0) no intercept	-0.64 (-2.3 to 0.99)
Quintile 4	229.1	218.6	-4.6% (-10.5% to 1.5%)	(2,1 12,0) no intercept	0.61 (-2.5 to 3.7)
Quintile 5 (highest)	236.4	237.2	0.4% (-5.7 to 6.6%)	(2,1 12,0) no intercept	1.5 (-1.3 to 4.3)
**Rural versus urban residence**					
Rural	229.6	233.1	1.5% (-7.6 to 11.1%)	(0,1 12,3) no intercept	-0.24 (-2.3 to 2.8)
Urban	243.4	232.4	-4.6% (-7.4% to -1.7%)	(2,1 12,0) no intercept	0.23 (-1.5 to 1.9)

## Discussion

In our population-based study, we observed an increase in the rate of benzodiazepine dispensing among children and youth following the implementation of a publicly-funded pharmacare program covering all prescription drug costs for Ontario residents between the ages of 0 and 24. Increased benzodiazepine dispensing was most pronounced among females, children and youth living in lower-income neighbourhoods, and individuals aged 15 to 19 and 20 to 24 years. Modifying the program to maintain coverage only for individuals lacking private insurance was not associated with a significant change in benzodiazepine dispensing trends.

Our findings add to previous research. As in other jurisdictions, we observed increased benzodiazepine dispensing over time, with greater use in females and older youth relative to younger children [4–7]. These findings reflect known patterns in the diagnosis of anxiety disorder in children and youth, with a higher prevalence among females and a typical age of diagnosis between early adolescence and young adulthood [31–33]. However, our study is the first to estimate the influence of a newly-implemented pharmacare program on benzodiazepine dispensing. Our finding of greater than expected benzodiazepine dispensing in the 12 months following the implementation of OHIP + is especially salient, highlighting the relative increase in benzodiazepene use attributable to this program. Moreover, we were able to study the differential impact of such a program on benzodiazepine use in specific sub-populations of children and youth. Therefore, our work extends the study of trends in benzodiazepine use among children and youth to a setting with no financial barriers to drug therapy.

Our study has important policy and practice implications for children and youth with mental health conditions. The largest increase in dispensing was in 20 to 24-year-olds, the group most commonly prescribed these drugs prior to the implementation of OHIP+. Although considered in the definition of youth, individuals aged to 20 to 24 can arguably be regarded as young adults for whom benzodiazepine medications need to be dispensed with all the cautions given to older adults. This age group includes individuals enrolled at post-secondary institutions with school insurance plans that typically cover only a portion of drug costs or place caps on annual prescription drug benefits. However, the increased dispensing of these drugs to adolescents aged 15 to 19 years immediately following the implementation of OHIP + is of concern, given the absence of evidence supporting long-term effectiveness for anxiety or sleep disorders and the potential for serious adverse effects, including decreased alertness, dependence, withdrawal, diversion and injury [34–36]. Moreover, there is concern that increased use of these drugs in children and youth could lead to a public health crisis similar to that observed with opioids [4, 37, 38]. For these reasons, benzodiazepines are not recommended for treating pediatric anxiety or sleep disorders and are considered second-line or short-term adjunctive therapies for adults [11, 12, 39–42]. Furthermore, although 4.0% of children and youth dispensed a benzodiazepine had a diagnosis of seizure disorder, the finding that nearly 1 in 5 individuals were dispensed a supply of benzodiazepines exceeding 30 days suggests long-term use for mental health conditions and symptoms could also be occurring. This practice is inconsistent with clinical guidelines advising short-term therapy of mental health symptoms until evidence-based therapies have been accessed [12]. Notably, most benzodiazepine prescriptions were written by general practitioners who may be less familiar with guidelines or have limited access to colleagues with expertise in treating pediatric mental health conditions. Increased use of telemedical health programs facilitating access to pediatric mental health specialists represents a promising mechanism for supporting general practitioners caring for children and youth with mental health conditions and optimizing psychotropic prescribing in this population [43, 44].

In addition, we observed a greater relative increase in benzodiazepine dispensing among children and youth in lower-income neighbourhoods following the implementation of OHIP+. This finding may reflect inequities created by long wait times for publicly-funded mental health and behavioural programs and out-of-pocket costs associated with accessing privately-funded interventions not covered by Ontario’s publicly-funded healthcare program, such as clinical psychologists and social workers [45–47]. A similar phenomenon may account for the greater immediate increase in benzodiazepine dispensing to children and youth in rural communities relative to urban centres following the implementation of OHIP+, where delays are incurred because of local service gaps and the travel costs associated with accessing care [45]. In contrast, there are no delays associated with accessing medications, and prescription drugs were available at no cost for all children and youth immediately following the implementation of OHIP+. It is therefore possible that systemic disparities in access to non-drug therapies create conditions that promote disproportionate reliance on benzodiazepines for symptom management among lower-income children and youth and individuals living in rural communities.

Strengths of our study include complete benzodiazepine dispensing data for all children and youth in Ontario, regardless of insurance status. However, our study has some limitations. First, we cannot reliably ascertain the appropriateness of benzodiazepine use or indication for therapy. This limitation is common to all population-based studies using claims-based data to study benzodiazepine use. Second, we could not determine whether benzodiazepines were dispensed for as-needed or ongoing use, or the proportion of children and youth progressing to long-term treatment with these drugs. Similarly, it is likely that a portion of children and youth dispensed a short supply of benzodiazepines were receiving these drugs for procedural anxiety, a practice which is generally well tolerated and does not involve continued use [48]. Third, it is possible that the period following the modification of OHIP + was too brief to detect a significant change. Fourth, misclassification of individuals with certain mental health diagnoses is possible because we used previously derived administrative algorithms that were not validated for our study [49]. Fifth, we did not longitudinally examine whether OHIP + changed the prevalence of long-term (e.g., 180 days) benzodiazepine use. Finally, our study was conducted in a single Canadian province, potentially limiting the generalizability of our findings. However, ours is the first population-based natural experiment study estimating the impact of a newly introduced pharmacare program on benzodiazepine dispensing among children and youth. Our findings suggest that additional measures may be needed in jurisdictions contemplating similar interventions to mitigate increases in benzodiazepine use, including improved access to behavioural and mental health interventions and measures to facilitate collaboration between primary care providers and pediatric mental health specialists.

In conclusion, implementing a publicly-funded pharmacare program was associated with increased benzodiazepine dispensing to children and youth and greater than expected use of these drugs. Further, we found disproportionate increases in the rate of benzodiazepine dispensing among females, adolescents and young adults and low-income children and youth. Although these trends align with the patterns of anxiety diagnosis in adolescents and young adults, socioeconomic differences in dispensing suggest that removing financial barriers to benzodiazepines may have increased the use of these drugs in a population with structural barriers to accessing guideline-recommended non-pharmacologic treatments. Moreover, past research in adults has found an association between increasing use of benzodiazepines, long-term use, misuse and benzodiazepine-related toxicity with low-income status or residence in low-income neighbourhoods [9, 50, 51, 52]. Further research is needed to determine if these associations extend to children and youth and to examine the role of alternative healthcare delivery models, such as telehealth and programs training primary care physicians to provide specialist care, is warranted to determine whether such interventions can improve prescribing. Moreover, interventions to support increased and equitable access to non-pharmacologic mental health expertise are required to minimize reliance on benzodiazepines for the treatment of mental health conditions in children and youth.

### Electronic supplementary material

Below is the link to the electronic supplementary material.

Supplementary Material 1

Supplementary Material 2

Supplementary Material 3

## Data Availability

The datasets generated and/or analysed during the current study are not publicly available due to data sharing agreements which prohibit ICES from making the data set publicly available. The data set from this study is held securely in coded form at ICES, and access may be granted to those who meet pre-specified criteria for confidential access, available at www.ices.on.ca/DAS. The full data set creation plan and underlying analytic code are available from the authors upon request (contact Dr. Tony Antoniou), understanding that the programs may rely upon coding templates or macros that are unique to ICES.

## Abbreviations

ARIMA: Autoregressive integrated moving average
Cis: Confidence intervals
IQR: Interquartile range
SD: Standardized difference
