# Supplementary material for: Impact of a publicly-funded pharmacare program policy on benzodiazepine dispensing among children and youth: a population-based natural experiment
Source: BMC Pediatr. 2023 Oct 19;23:519. doi: 10.1186/s12887-023-04331-4 (PMC10585894; doi:10.1186/s12887-023-04331-4)

**Supplemental Figures**

Figure 1: Impact of OHIP+ implementation (January 2018) and modification (April 2019) on monthly rates of benzodiazepine dispensing among Ontario residents between the ages of 0 and 24, stratified by sex, January 2013 to March 2020


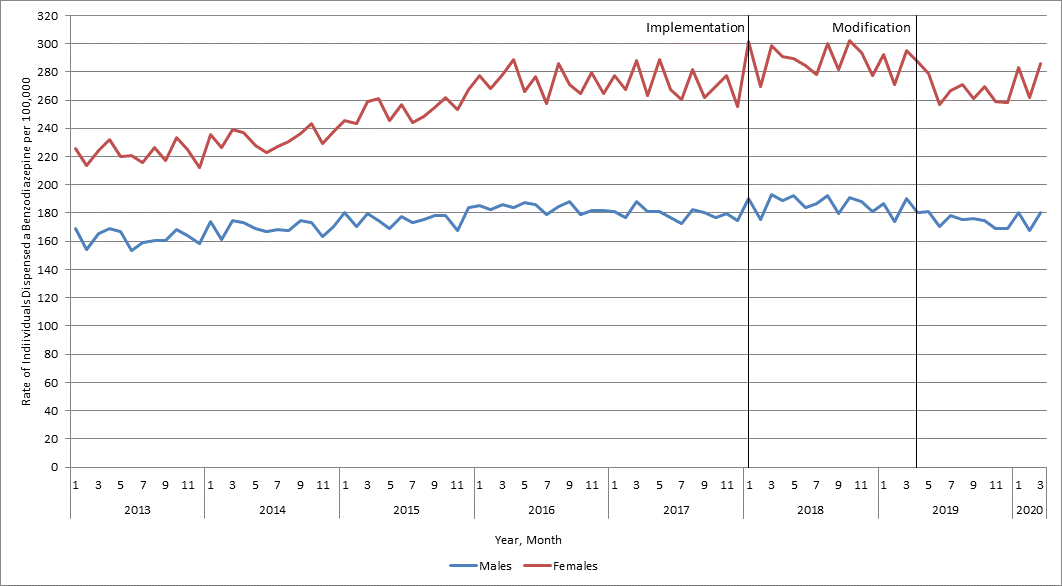


Figure 2: Impact of OHIP+ implementation (January 2018) and modification (April 2019) on monthly rates of benzodiazepine dispensing among Ontario residents between the ages of 0 and 24, stratified by age, January 2013 to March 2020


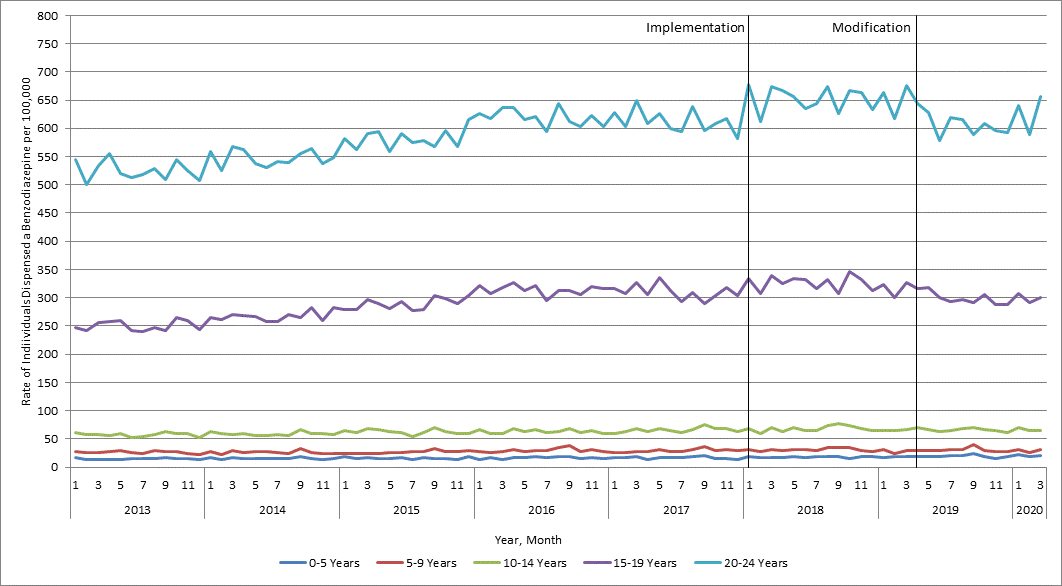


Figure 3: Impact of OHIP+ implementation (January 2018) and modification (April 2019) on monthly rates of benzodiazepine dispensing among Ontario residents between the ages of 0 and 24, stratified by neighbourhood income quintile (quintile 1 = lowest), January 2013 to March 2020


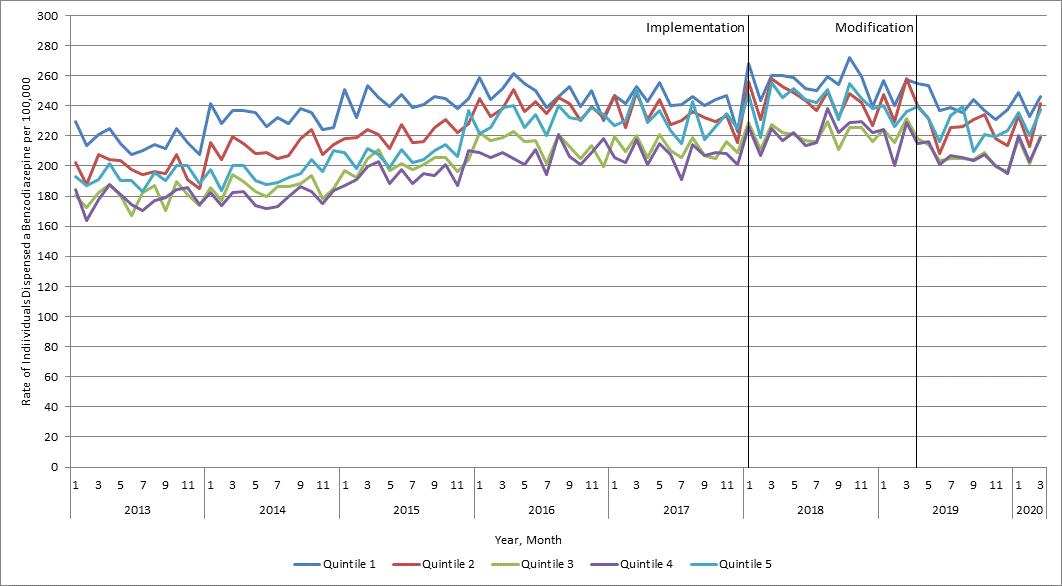


Figure 4: Impact of OHIP+ implementation (January 2018) and modification (April 2019) on monthly rates of benzodiazepine dispensing among Ontario residents between the ages of 0 and 24, stratified by urban versus rural residence, January 2013 to March 2020


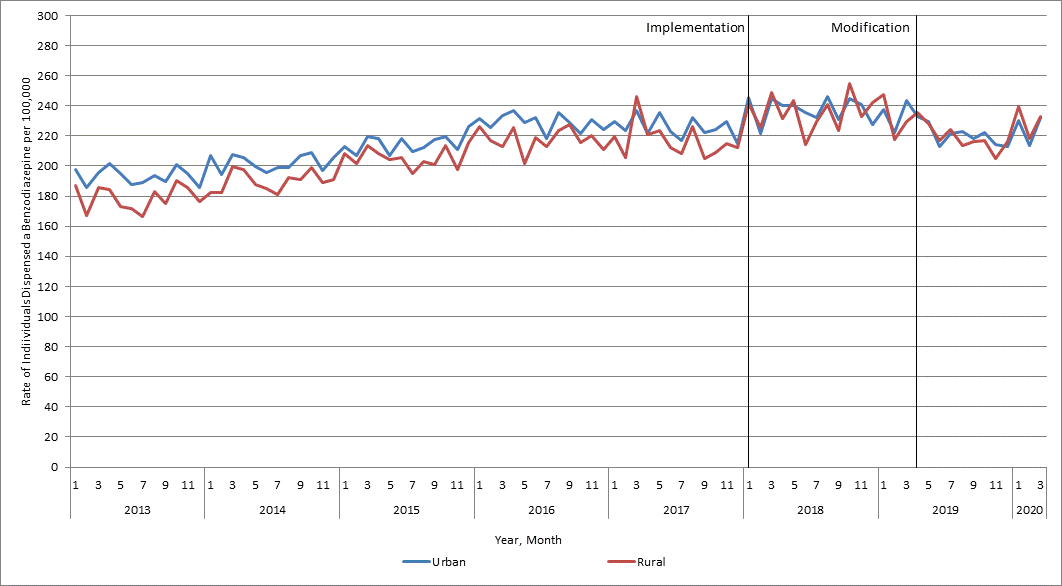

Supplement: Supplementary file 1 — Supplementary Material 1 [file 12887_2023_4331_MOESM1_ESM.docx]
