# Supplementary material for: Impact of a publicly-funded pharmacare program policy on benzodiazepine dispensing among children and youth: a population-based natural experiment
Source: BMC Pediatr. 2023 Oct 19;23:519. doi: 10.1186/s12887-023-04331-4 (PMC10585894; doi:10.1186/s12887-023-04331-4)
Supplement: Supplementary file 2 — Supplementary Material 2 [file 12887_2023_4331_MOESM2_ESM.docx]

**Supplemental Table 1: Diagnosis Codes Associated with Mental Health Clinical Categories**

| **Clinical category** | **OHIP diagnostic codes (OHIP)^a^** | **DSM-IV (OMHRS)^a^** | **ICD-10-CA codes (DAD/NACRS)^a^** |
| --- | --- | --- | --- |
| **Mood disorders** | 296, 311 | 296.x (all 296 codes), 300.4x, 301.13, 311.x | F30, F31, F32, F33, F34, F38, F39, F53.0 |
| **Anxiety disorders** | 300 | 300, 300.0x, 300.2x, 300.3x, 308.3x, 309.0x, 309.24, 309.28, 309.3x, 309.4x, 309.8x, 309.9x. | F40, F41, F42, F43, F48.8, F48.9; F93.1, F93.2 |
| **Seizure disorders** | 345 |  | G40, G41, G56 |

^a^OHIP = Ontario Health Insurance Plan, OMHRS=Ontario Mental Health Reporting System, DAD=Discharge Abstract Database, NACRS=National Ambulatory Care Reporting System

**Supplemental Table 2: Demographic characteristics according to whether individuals dispensed less than or greater than 30 days’ supply of benazodiazepine**

| **Variable^a^** | **Entire Study Period (January 1, 2013 to March 31, 2020)** | | **Pre-OHIP+ (January 1, 2017 to December 31, 2017)** | | **During OHIP+ (January 1, 2018 to March 30, 2019)** | | **Post-OHIP+ (April 1, 2019 to March 31, 2020)** | |
| --- | --- | --- | --- | --- | --- | --- | --- | --- |
|  | **<30 days’ supply** | **>30 days’ supply** | **<30 days’ supply** | **>30 days’s supply** | **<30 days’ supply** | **>30 days’ supply** | **<30 days’ supply** | **>30 days’ supply** |
| Number of individuals | 182,877 | 35,411 | 39,073 | 10,800 | 50,060 | 11,976 | 39,959 | 9,675 |
| Age (median, IQR) | 20 (17-22) | 20 (17-22) | 20 (17-22) | 21 (18-23) | 20 (17-22) | 21 (18-23) | 20 (17-22) | 21 (18-23) |
| 0-4 | 3,853 (2.1%) | 426 (1.2%) | 715 (1.8%) | 101 (0.9%) | 1,008 (2.0%) | 120 (1.0%) | 886 (2.2%) | 119 (1.2%) |
| 5-9 | 5,961 (3.3%) | 680 (1.9%) | 1,287 (3.3%) | 218 (2.0%) | 1,619 (3.2%) | 252 (2.1%) | 1,402 (3.5%) | 215 (2.2%) |
| 10-14 | 14,711 (8.0%) | 2,420 (6.8%) | 2,917 (7.5%) | 597 (5.5%) | 3,706 (7.4%) | 713 (6.0%) | 3,113 (7.8%) | 562 (5.8%) |
| 15-19 | 64,906 (35.5%) | 12,074 (34.1%) | 12,450 (31.9%) | 3,159 (29.3%) | 15,938 (31.8%) | 3,406 (28.4%) | 12,553 (31.4%) | 2,716 (28.1%) |
| 20-24 | 93,446 (51.1%) | 19,811 (55.9%) | 21,704 (55.5%) | 6,725 (62.3%) | 27,789 (55.5%) | 7,485 (62.5%) | 22,005 (55.1%) | 6,063 (62.7%) |
| Female, No. (%) | 114,605 (62.7%) | 21,480 (60.7%) | 24,857 (63.6%) | 6,665 (61.7%) | 31,891 (63.7%) | 7,492 (62.6%) | 25,405 (63.6%) | 6,026 (62.3%) |
| Income quintile |  |  |  |  |  |  |  |  |
| 1 (lowest) | 34,706 (19.0%) | 7,603 (21.5%) | 7,620 (19.5%) | 2,243 (20.8%) | 9,678 (19.3%) | 2,430 (20.3%) | 7,711 (19.3%) | 1,976 (20.4%) |
| 2 | 33,891 (18.5%) | 6,766 (19.1%) | 7,228 (18.5%) | 2,096 (19.4%) | 9,137 (18.3%) | 2,424 (20.2%) | 7,320 (18.3%) | 1,814 (18.7%) |
| 3 | 35,011 (19.1%) | 6,661 (18.8%) | 7,413 (19.0%) | 2,062 (19.1%) | 9,639 (19.3%) | 2,239 (18.7%) | 7,635 (19.1%) | 1,860 (19.2%) |
| 4 | 37,422 (20.5%) | 6,956 (19.6%) | 7,815 (20.0%) | 1,998 (18.5%) | 10,282 (20.5%) | 2,291 (19.1%) | 8,163 (20.4%) | 1,918 (19.8%) |
| 5 | 41,847 (22.9%) | 7,425 (21.0%) | 8,997 (23.0%) | 2,401 (22.2%) | 11,324 (22.6%) | 2,592 (21.6%) | 9,130 (22.8%) | 2,107 (21.8%) |
| Residence |  |  |  |  |  |  |  |  |
| Urban | 164,569 (90.0%) | 32,076 (90.6%) | 35,326 (90.4%) | 9,827 (91.0%) | 45,114 (90.1%) | 10,860 (90.7%) | 35,890 (89.8%) | 8,851 (91.5%) |
| Rural | 18,308 (10.0%) | 3,335 (9.4%) | 3,747 (9.6%) | 973 (9.0%) | 4,946 (9.9%) | 1,116 (9.3%) | 4,069 (10.2%) | 824 (8.5%) |
| Prescriber Type |  |  |  |  |  |  |  |  |
| General Practitioner | 115,165 (63.0%) | 20,053 (56.6%) | 24,503 (62.7%) | 5,914 (54.8%) | 30,873 (61.7%) | 6,507 (54.3%) | 24,270 (60.7%) | 5,018 (51.9%) |
| Pediatrician | 9,134 (5.0%) | 1,336 (3.8%) | 2,098 (5.4%) | 508 (4.7%) | 2,673 (5.3%) | 523 (4.4%) | 2,247 (5.6%) | 442 (4.6%) |
| Psychiatrist | 20,930 (11.4%) | 10,933 (30.9%) | 5,604 (14.3%) | 3,535 (32.7%) | 7,007 (14.0%) | 3,946 (32.9%) | 5,681 (14.2%) | 3,293 (34.0%) |
| Other | 37,648 (20.6%) | 3,089 (8.7%) | 6,868 (17.6%) | 843 (7.8%) | 9,507 (19.0%) | 1,000 (8.4%) | 7,761 (19.4%) | 922 (9.5%) |
| Diagnosis in 30 days preceding dispensing date |  |  |  |  |  |  |  |  |
| Anxiety disorder | 84,966 (46.5%) | 17,427 (49.2%) | 17,361 (44.4%) | 4,387 (40.6%) | 22,366 (44.7%) | 5,008 (41.8%) | 17,300 (43.3%) | 3,745 (38.7%) |
| Mood disorder | 20,197 (11.0%) | 6,401 (18.1%) | 4,580 (11.7%) | 1,639 (15.2%) | 5,900 (11.8%) | 1,926 (16.1%) | 4,488 (11.2%) | 1,533 (15.8%) |
| Seizure disorder | 7,828 (4.3%) | 988 (2.8%) | 1,765 (4.5%) | 293 (2.7%) | 2,400 (4.8%) | 373 (3.1%) | 2,231 (5.6%) | 340 (3.5%) |

**Supplemental Table 3: Most common benzodiazepines dispensed, stratified by age (< 12 years versus > 12 years)**

| **Variable^a^** | **Entire Study Period (January 1, 2013 to March 31, 2020)** | | **Pre-OHIP+ (January 1, 2017 to December 31, 2017)** | | **During OHIP+ (January 1, 2018 to March 30, 2019)** | | **Post-OHIP+ (April 1, 2019 to March 31, 2020)** | |
| --- | --- | --- | --- | --- | --- | --- | --- | --- |
|  | **<12 years** | **>12 years** | **<12 years** | **>12 years** | **<12 years** | **>12 years** | **<12 years** | **>12 years** |
| Number of prescriptions | 47,752 | N=1,137,298 | N=6,762 | N=163,365 | N=8,888 | N=217,612 | N=7,258 | N=164,638 |
| Clonazepam | 13,375 (28.0%) | 483,653 (42.5%) | 1,852 (27.4%) | 70,493 (43.2%) | 2,140 (24.1%) | 91,585 (42.1%) | 1,988 (27.4%) | 67,861 (41.2%) |
| Diazepam | 4,237 (8.9%) | 67,926 (6.0%) | 683 (10.1%) | 9,956 (6.1%) | 810 (9.1%) | 13,931 (6.4%) | 578 (8.0%) | 12,106 (7.4%) |
| Lorazepam | 26,995 (56.5%) | 485,967 (42.7%) | 3,876 (57.3%) | 69,169 (42.3%) | 5,440 (61.2%) | 94,477 (43.4%) | 4,357 (60.0%) | 72,593 (44.1%) |
| Other^*^ | 3145 (6.6%) | 99,752 (8.8%) | 351 (5.2%) | 13,747 (8.4%20 | 498 (5.6%) | 17,619 (8.1%) | 335 (4.6%) | 12,078 (7.3%) |

^*^Includes: alprazolam, bromazepam, chlordiazepoxide, clorazepate, flurazepam, nitrazepam, oxazepam, temazepam, triazolam

**Supplemental Table 4a: Most common benzodiazepines dispensed by neighbourhood income quintile over entire study period (January 1, 2013 to March 31, 2020)**

|  | Income quintile 1 (lowest) | Income quintile 2 | Income quintile 3 | Income quintile 4 | Income quintile 5 (highest) |
| --- | --- | --- | --- | --- | --- |
| Number of prescription claims | 301,445 | 232,124 | 209,395 | 211,295 | 230,791 |
| Alprazolam | 9,785 (3.2%) | 8,173 (3.5%) | 7,042 (3.4%) | 8,856 (4.2%) | 9,932 (4.3%) |
| Clonazepam | 138,334 (45.9%) | 102,167 (44.0%) | 85,023 (40.6%) | 81,829 (38.7%) | 89,675 (38.9%) |
| Diazepam | 22,014 (7.3%) | 13,933 (6.0%) | 13,141 (6.3%) | 12,270 (5.8%) | 10,805 (4.7%) |
| Lorazepam | 114,545 (38.0%) | 96,383 (41.5%) | 93,735 (44.8%) | 98,632 (46.7%) | 109,667 (47.5%) |
| Other^*^ | 16,767 (5.6%) | 11468 (4.9%) | 10,454 (5.0%) | 9,708 (4.6%) | 10,712 (4.6%) |

**Supplemental Table 4b: Most common benzodiazepines dispensed by neighbourhood income quintile, pre-OHIP+ period (January 1, 2017 to December 31, 2017)**

|  | Income quintile 1 (lowest) | Income quintile 2 | Income quintile 3 | Income quintile 4 | Income quintile 5 (highest) |
| --- | --- | --- | --- | --- | --- |
| Number of prescription claims | **44,219** | **32,849** | **29,035** | **29,753** | **34,271** |
| Alprazolam | 1,440 (3.3%) | 1,269 (3.9%) | 1,091 (3.8%) | 1,400 (4.7%) | 1,444 (4.2%) |
| Clonazepam | 20,141 (45.5%) | 14,920 (45.4%) | 11,644 (40.1%) | 11,829 (39.8%) | 13,811 (40.3%) |
| Diazepam | 4,389 (9.9%) | 1,669 (5.1%) | 1,412 (4.9%) | 1,736 (5.8%) | 1,433 (4.2%) |
| Lorazepam | 16,212 (36.7%) | 13,624 (41.5%) | 13,416 (46.2%) | 13,481 (45.3%) | 16,312 (47.6%) |
| Other^*^ | 2,037 (4.6%) | 1,367 (4.2%) | 1,472 (5.1%) | 1,307 (4.4%) | 1,271 (3.7%) |

^*^Includes: bromazepam, chlordiazepoxide, clorazepate, flurazepam, nitrazepam, oxazepam, temazepam, triazolam

**Supplemental Table 4c: Most common benzodiazepines dispensed by neighbourhood income quintile, OHIP+ period (January 1, 2018 to March 31, 2019)**

|  | Income quintile 1 (lowest) | Income quintile 2 | Income quintile 3 | Income quintile 4 | Income quintile 5 (highest) |
| --- | --- | --- | --- | --- | --- |
| Number of prescription claims | **54,944** | **43,548** | **40,324** | **41,963** | **45,721** |
| Alprazolam | 1,793 (3.3%) | 1,392 (3.2%) | 1,381 (3.4%) | 2,437 (5.8%) | 2,273 (5.0%) |
| Clonazepam | 23,990 (43.7%) | 19,590 (45.0%) | 15,996 (39.7%) | 16,145 (38.5%) | 18,004 (39.4%) |
| Diazepam | 4,498 (8.2%) | 2,468 (5.7%) | 3,340 (8.3%) | 2,200 (5.2%) | 2,235 (4.9%) |
| Lorazepam | 22,256 (40.5%) | 18,354 (42.1%) | 18,023 (44.7%) | 19,693 (46.9%) | 21,591 (47.2%) |
| Other^*^ | 2,407 (4.4%) | 1,744 (4.0%) | 1,584 (3.9%) | 1,488 (3.5%) | 1,618 (3.5%) |

**Supplemental Table 4d: Most common benzodiazepines dispensed by neighbourhood income quintile, post-OHIP+ period (April 1, 2019 to March 31, 2020)**

|  | Income quintile 1 (lowest) | Income quintile 2 | Income quintile 3 | Income quintile 4 | Income quintile 5 (highest) |
| --- | --- | --- | --- | --- | --- |
| Number of prescription claims | **N=41,113** | **N=34,019** | **N=31,233** | **N=32,112** | **N=33,419** |
| Alprazolam | 1,163 (2.8%) | 1,359 (4.0%) | 848 (2.7%) | 1,156 (3.6%) | 1,337 (4.0%) |
| Clonazepam | 18,746 (45.6%) | 13,767 (40.5%) | 12,568 (40.2%) | 12,200 (38.0%) | 12,568 (37.6%) |
| Diazepam | 2,952 (7.2%) | 2,853 (8.4%) | 2,352 (7.5%) | 2,501 (7.8%) | 2,026 (6.1%) |
| Lorazepam | 16,575 (40.3%) | 14,719 (43.3%) | 14,499 (46.4%) | 14,945 (46.5%) | 16,212 (48.5%) |
| Other^*^ | 1,677 (4.1%) | 1,321 (3.9%) | 966 (3.1%) | 1,310 (4.1%) | 1,276 (3.8%) |

*Includes: bromazepam, chlordiazepoxide, clorazepate, flurazepam, nitrazepam, oxazepam, temazepam, triazolam
