# Supplementary material for: Impact of a publicly-funded pharmacare program policy on benzodiazepine dispensing among children and youth: a population-based natural experiment
Source: BMC Pediatr. 2023 Oct 19;23:519. doi: 10.1186/s12887-023-04331-4 (PMC10585894; doi:10.1186/s12887-023-04331-4)

Thursday, August 31, 2023

To whom it may concern:

**NOTICE re: ICES Projects Conducted Under Section 45 of PHIPA**

ICES is a prescribed entity under section 45 of *Ontario’s Personal Health Information Protection Act* (PHIPA). Section 45 is the provision that enables analysis and compilation of statistical information related to the management, evaluation and monitoring of, allocation of resources to, and planning for the health system. Section 45 authorizes health information custodians to disclose personal health information to a prescribed entity, like ICES, without consent for such purposes.

Projects conducted wholly under section 45, by definition, do not require review by a Research Ethics Board. This is confirmed in a letter from the REB of Sunnybrook Health Sciences Centre, ICES’ Research Ethics Board of Record (See Appendix A).

As a prescribed entity, ICES must submit to tri-annual review and approval of its privacy and security policies, procedures and practices by Ontario’s Information and Privacy Commissioner. These include policies, practices and procedures that require internal review and approval of every project by ICES’ Privacy and Compliance Office. ICES was approved by the Commissioner for a fifth time in 2017.

Please do not hesitate to contact us if you require further information.

Sincerely,

ICES

Privacy & Legal Office

G-106, 2075 Bayview Avenue, Toronto, ON M4N 3M5

T: 416-480-4055

F: 416-480-6048

[plo@ices.on.ca](mailto:plo@ices.on.ca)

Appendix A


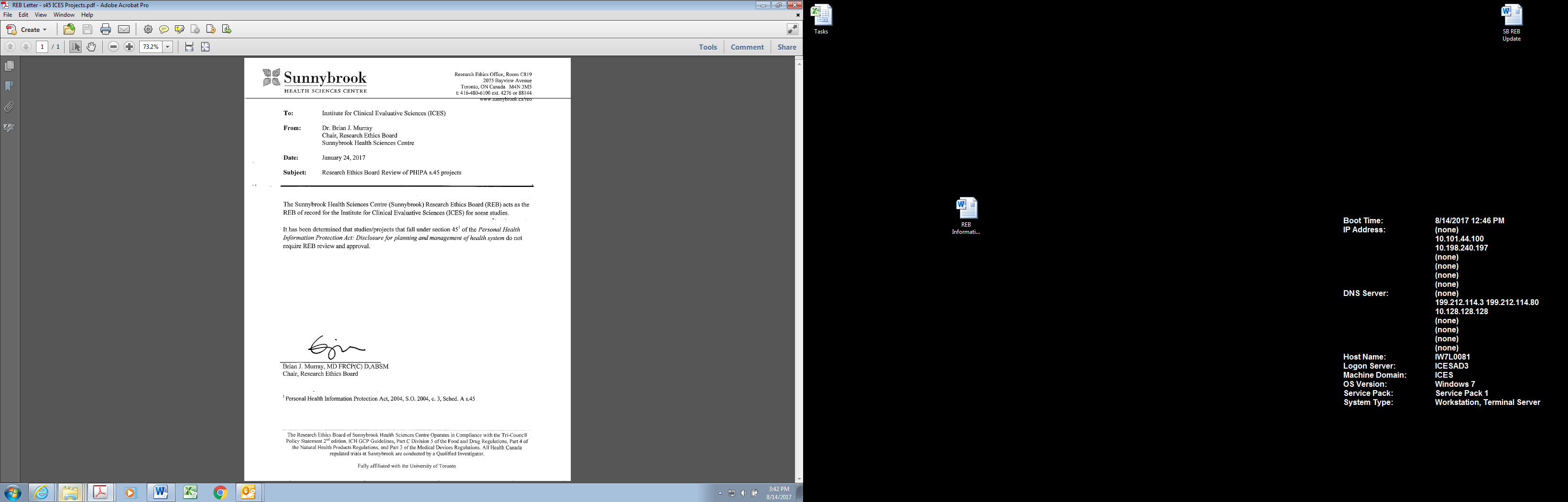

Supplement: Supplementary file 3 — Supplementary Material 3 [file 12887_2023_4331_MOESM3_ESM.docx]
